# Effects of Interval Exercise Training on Serum Biochemistry and Bone Mineral Density in Dogs

**DOI:** 10.3390/ani11092528

**Published:** 2021-08-28

**Authors:** Hae Sung Lee, Jae Hwan Kim, Hyun Ju Oh, Jong Hee Kim

**Affiliations:** 1 Department of Physical Education, College of Performing Arts and Sport, Hanyang University, 222 Wangsimni-ro, Seongdong-gu, Seoul 04763, Korea; hsleee@hanyang.ac.kr; 2 Department of Veterinary Medical Imaging, College of Veterinary Medicine, Konkuk University, 120 Neungdong-ro, Gwangjin-gu, Seoul 05029, Korea; jaehwan@konkuk.ac.kr; 3 Department of Animal Sciences and Biotechnology, College of Agriculture and Life Science, Chungnam National University, 99 Daehak-ro, Yuseong-gu, Daejeon 34134, Korea

**Keywords:** interval exercise, exercise physiology, bone mineral density, immune function

## Abstract

**Simple Summary:**

In this study, six male beagle dogs underwent 12 weeks of interval exercise following the Frequency, Intensity, Time/duration, Type, Volume, and Progression (FITT-VP) training principle. The heart rate (HR) response was measured during the entire exercise period, and changes in bone mineral density (BMD), muscle volume (MV), and hematology and serum biomarkers were evaluated at the pre-exercise training period and post-exercise training period. We showed that exercise training increased BMD in the femur and serum total alkaline phosphatase (TALP), aspartate aminotransferase, and creatine kinase levels. In addition, our data suggest a positive correlation between BMD and TALP, demonstrating that increased TALP might be an important contributing factor for enhancing BMD with physical training in dogs.

**Abstract:**

Exercise has been suggested as a powerful intervention for health care and fitness management in humans; however, few studies have demonstrated the benefits of exercise training in dogs. The purpose of this study was to examine the effects of exercise training on heart rate (HR), bone mineral density (BMD), muscle volume (MV), and hematological and serum biomarkers in dogs. Six healthy beagles completed the interval treadmill exercise, developed on the basis of the FITT principle, two times a week for 12 weeks. To evaluate the physiological parameters, the HR values were analyzed using the Polar H10 system during the entire exercise period. At pre-and post-exercise, quantitative computed tomography and hematological and serum biochemical parameters were analyzed. The interval exercise resulted in a normal HR response and no adverse behavioral or physiological effects on the dogs. We showed that exercise improved BMD in the femur (541.6 ± 16.7 vs. 610.2 ± 27.8 HA, *p* < 0.01) and increased serum total alkaline phosphatase (TALP; 68.6 ± 9.2 vs. 81.3 ± 17.2, *p* < 0.01), aspartate aminotransferase (23.5 ± 1.0 vs. 33.5 ± 1.6, *p* < 0.01), and creatine kinase (114.8 ± 5.3 vs. 214.0 ± 20.8, *p* < 0.01) levels. There was a positive relationship between BMD and TALP (femur: r = 0.760, *p* = 0.004; vertebrae: r = 0.637; *p* = 0.025). Our findings suggest that interval exercise training is beneficial to increase BMD in the femur, and an increased TALP level would be a concomitant mechanism for enhancing BMD with exercise in dogs.

## 1. Introduction

Dogs, a common companion animal, have coexisted with humans since the Neolithic Age and are recognized as family members in many modern societies [1]. Studies have reported that over 70% of dog owners feel affection for their canine companions akin to raising a baby [2]. Additionally, many different types of working or service dogs were bred to help people accomplish day-to-day tasks [3]. As the bonds between dogs and humans grow more substantial, there is greater demand for better canine health care and fitness management [1]. Exercise has been recognized as an essential component for attaining optimal health and fitness levels [2,4]. Many human studies have robustly established that exercise training improves health-related fitness [5,6], but research exploring the benefits of the exercise training system in dogs is limited.

Studies have shown that physiological and biochemical parameters are fundamental to investigate the effects of exercise on health, fitness, and function [7,8,9,10]. Heart rate (HR) [10] is a primary physiological indicator for diagnosing cardiac function and aerobic performance. Exercise-induced adaptation in HR (i.e., reduction in resting HR) is related to improved cardiovascular fitness [9]. HR response during competitive exercise and recovery can also be used to assess heart-rhythm disorders, such as arrhythmia, in dogs [9]. HR levels influence cardiac output and VO_2max_ during exercise [10]. In addition, HR responses, which are known to reflect the complex interactions between the autonomic nervous system and the cardiovascular system, provide significant prognostic information. Inadequate HR response to exercise is strongly associated with sudden cardiac death, and HR recovery on a standard exercise test has been shown to predict mortality [11]. Hematological and serum biochemical analyses can reveal systemic and metabolic functions. Treadmill exercise studies showed significant improvements in rectal temperature, glucose and lactate concentrations, red blood cell counts, hematocrit, and HR in dogs [12,13]. Although each parameter has its benefits, studies carrying out comprehensive examinations required for a thorough screening of a dog’s health are limited, and the effects of long-term exercise-induced adaptation in those parameters are still elusive.

Bone mineral density (BMD) and muscle volume (MV) are valuable measurements to assess overall health status and bone-muscle interactions in post-exercise dogs. Some human studies have found that low BMD and MV are associated with several disorders, including inflammatory diseases, osteogenesis imperfecta, degenerative arthritis, and endocrine diseases [14,15]. Furthermore, exercise (e.g., sprinting, jogging, weightlifting, swimming) can be a powerful intervention for the prevention and treatment of these conditions in humans [16,17,18]. In several other studies, participants of different ages and genders showed positive changes in BMD, MV, and bone turnover serum biomarkers (e.g., bone-specific alkaline phosphatase, deoxypyridinoline, and calcium) after exercise designed according to the Frequency, Intensity, Time/duration, Type, Volume, and Progression (FITT-VP) principle [19,20,21,22,23]. To date, however, there has been little scientific examination of the effect of exercise on bone and muscle health in dogs. One reason for this lack of knowledge is the ethical issue of studying bone and muscle tissues, which requires invasive procedures.

Non-invasive quantitative computed tomography (QCT) is a three-dimensional non-projection technique to evaluate BMD and MV. In human studies, QCT has been used to investigate muscle-bone interactions [24,25]. In veterinary medicine, however, QCT is mainly used to diagnose skeletal changes in aging, osteoporosis, and other metabolic bone diseases [26,27,28,29,30,31]. For example, Sutherland-Smith and colleagues [30] exhibited age-induced epaxial muscle atrophy by using CT scanning in dogs. Gordon-Evans et al. [31] showed positive effects of physical rehabilitation on body fat and muscle mass using DEXA in dachshunds with thoracolumbar intervertebral disk disease. To date, there have been no studies using QCT to assess changes in BMD and MV after exercise in dogs, nor have there been studies that examined the relationship between biomarker levels and bone-muscle properties. We postulated that it would be possible to use QCT to assess subtle changes in BMD and MV after treadmill exercise training. Therefore, this study aimed to investigate the alterations in HR, blood, serum, BMD, and MV after performing an interval exercise protocol in dogs. We hypothesized that the 12-week interval exercise training would result in positive changes in HR, hematology, and serum biochemistry parameters and increase BMD and MV in healthy dogs.

## 2. Materials and Methods

### 2.1. Animals

Six healthy male beagles who had never experienced exercise training were included in the study and participated in the whole experiment. Information on these dogs is provided in Table 1. All dogs were cared for following the recommendations described in The Guide for the Care and Use of Laboratory Animals.

The study was approved by the Institutional Animal Care and Use Committee of Hanyang University and Seoul National University (HYU-2020-0073A, SNU-180731-2). All methods and protocols were carried out in accordance with the relevant guidelines and regulations. Moreover, all beagles were subject to the same dietary and resting conditions. The dogs were housed in an environment with 12 h (07:00–19:00) of bright light and 12 h (19:00–07:00) of dark. The temperature of the breeding room was 22–23 °C, with 50–60% humidity. The dogs were kept in separate cages (775 × 960 × 900 cm) with soft rubber flooring that was cleaned daily. Meals (Eco 1 LAMB, Natural core, Gyeonggi-do, Korea) were served twice a day (09:00, 17:00), and freshwater was provided freely. The diet was kept the same throughout the study period. The dogs were not provided with food for 4 h prior to exercise testing to prevent exercise-induced gastrointestinal distress, heartburn, and acid reflux.

### 2.2. Treadmill Adaptation for Dog Safety

All dogs underwent 2 weeks of adaptive training to be acquainted with the researcher, laboratory environment, and exercise regimen in advance (Supplementary Table S1). The exercise training equipment included a treadmill (EGOJIN XG-V6E, Gyeonggi-do, Korea) and a safety belt, which was applied to each dog’s chest. Rectal temperature was taken from each dog with a digital thermometer before and after exercise. Throughout the experiment, the researcher and veterinarian screened the dogs’ behavior and reactions (i.e., limp, a strong rejection of exercise, pupil abnormalities, and very rapid and irregular heart rhythms during exercise) to confirm the safety and determine any unexpected discontinuation of exercise.

### 2.3. Interval Exercise Program

**Figure 1:**
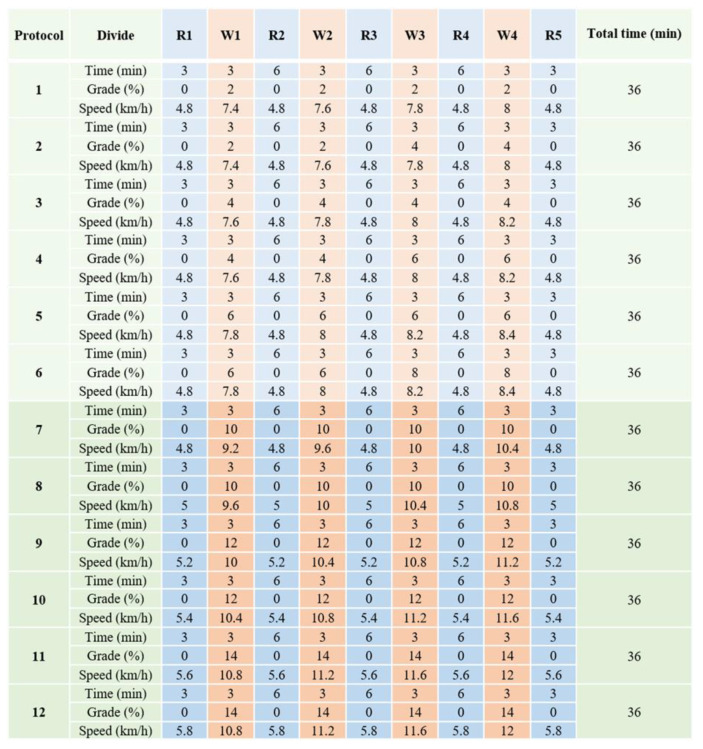
A 12-week interval exercise program consisting of 12 protocols. The 12 protocols include a gradual increase in grade (%) and speed (km/h) as the sessions proceed.

In this study, we modified the 12-week interval exercise protocol developed in our previous study [7]. As a warm-up, the dogs performed a walking exercise for 5 min at 2–3 km/h prior to interval exercise. The exercise training program consisted of 12 treadmill protocols, which are detailed in Figure 1. Each protocol was repeated twice per week for the numerically corresponding study week. The protocol consisted of a workout stage (W) and an incomplete resting stage (R). Exercise intensity was gradually increased by changing treadmill grade and speed.

### 2.4. Heart Rate Measurement

A Polar H10 HR measuring device and monitor (Polar Electro Oy, Kempele, Finland) were used to evaluate HR response during interval exercise. The dogs wore HR measuring devices on their chests, and HR data were collected every second. The mean HR value was analyzed to estimate exercise intensity for each stage in all protocols using the Polar Flow Software program (Polar Electro Oy, Kempele, Finland).

### 2.5. Quantitative Computed Tomography (Bone Mineral Density and Muscle Volume)

**Figure 2:**
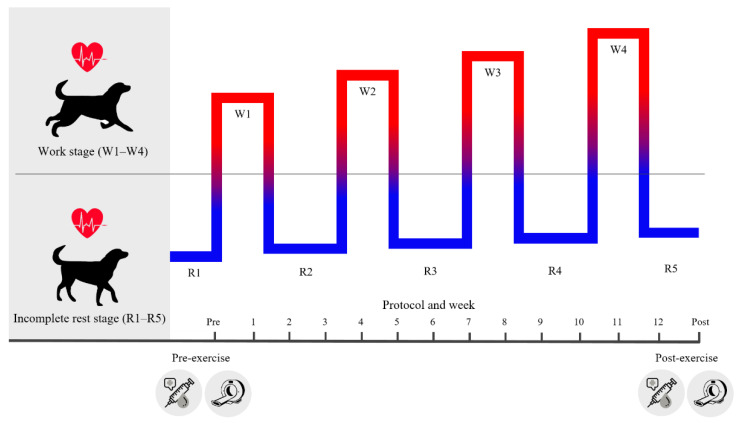
Schematic design of experimental procedures. Dogs underwent interval exercise comprising five resting stages and four workout stages over 12 weeks. Blood biochemical parameters (pre-exercise, post-exercise), heart rate (overall interval exercise), and QCT (pre-exercise, post-exercise) were measured for each dog.

QCT was used to measure BMD and MV. All dogs were fasted for at least 6 h prior to QCT scan. The dogs were intravenously premedicated with glycopyrrolate (Mobinul; Myungmoon Pharm., Seoul, Korea) at 0.01 mg/kg and then anesthetized with 6 mg/kg of propofol (Provive; Myungmoon Pharm., Seoul, Korea). They were kept sedated with 1.5% isoflurane (Foran solution; Choongwae Pharm., Seoul, Korea) and received 100% oxygen via endotracheal tube intubation. Percentage of oxygen saturation, end-tidal CO_2_, and HR were routinely monitored. QCT was performed using a 16-channel multidetecting CT scanner (Brivo 385; GE Medical System, Milwaukee, WI, USA). The lumbar vertebrae and femur were scanned with the dogs in dorsal recumbency. A calibrated QCT phantom (QRM-BDC/3; QRM GmbH, Moehrendorf, Germany) was placed under each dog. The scanning parameters were set as follows: 100 kV, 100 mAs, and 1.25-mm slice thickness, pitch 1.5:1, rotation time 0.6 sec, and scanning speed 7.5 mm/rotation. The phantom and femur were positioned such that their axes were perpendicular to each other and reconstructed in the transverse plane. The phantom and lumbar vertebrae were positioned parallel to each other. All QCT images were scanned with the bone and beam placed close to vertical without tilting of the gantry using the bone algorithm. CT scan was performed before and after exercise (Figure 2). All CT images were analyzed using commercially available software (RadiAnt DICOM viewer; Medixant, Poznan, Poland; Osirix DICOM viewer; Pixmeo, Geneva, Switzerland). The region of interest (ROI) for QCT included only the vertebral body in the 3rd lumbar vertebra and was measured using an image at the origin of the transverse process as previously described [32,33]. The cortical and trabecular bone at all measurement sites were included in the ROI. Femoral BMD was measured in the middle of the femoral neck, including one-third of the proximal diaphysis and one-third of the distal diaphysis. BMD was calculated from the CT image in Hounsfield units. MV was measured at the correct position using multi-planar reconstruction at 50% of the femur length, and a cross-section perpendicular to the bone was obtained. Each variable was measured three times, and the mean value for each was obtained.

### 2.6. Hematology and Serum Biochemistry Parameter Analysis

Blood samples for hematological and serum biochemical parameter analyses were collected the day before protocol 1 initiation and 1 day after protocol 12 completion (Figure 2). Blood samples were kept in tubes coated with lithium heparin and stored at 4 °C. After blood withdrawal and plasma harvest, heparinized blood samples were allowed to clot and were then centrifuged to obtain serum. All analyses were performed within the first 6 h after blood extraction. Hematological parameters were measured from EDTA-blood samples using ADVIA 2120i (NYN Tarrytown, Tarrytown, NY, USA). Biochemistry parameters were measured from heparinized plasma using the Hitachi 7180 Auto analyzer (Hitachi, Tokyo, Japan) with reagents specifically designed for the instrument.

### 2.7. Statistical Analyses

All analyses were performed with GraphPad Prism 5.0 (GraphPad Inc., La Jolla, CA, USA). A one-way repeated analysis of variance was used to determine the mean difference in HR, followed by a Bonferroni post-hoc test. During all processes of interval exercise, the HR data were collected on a per-second basis and sent from the Polar Beat app monitor (v:3.5.0) to the Polar Flow software (Polar Flow online: https://flow.polar.com/ (accessed on 16 August 2021)). The transmitted HR information was analyzed separately for the resting HR 1 min before exercise, the mean HR in each stage of exercise, and the recovery HR 1 min after exercise and until returning to the resting HR. The mean difference in BMD, MV, and serum biochemistry parameters between pre-exercise and post-exercise was assessed using a two-tailed Student’s t-test. To determine the relationship between total alkaline phosphatase (TALP) and BMD and MV, we performed Spearman’s correlation and linear regression analyses. Values are expressed as means ± SEMs, and a *p*-value < 0.05 was considered statistically significant.

## 3. Results

### 3.1. Heart Rate

**Figure 3:**
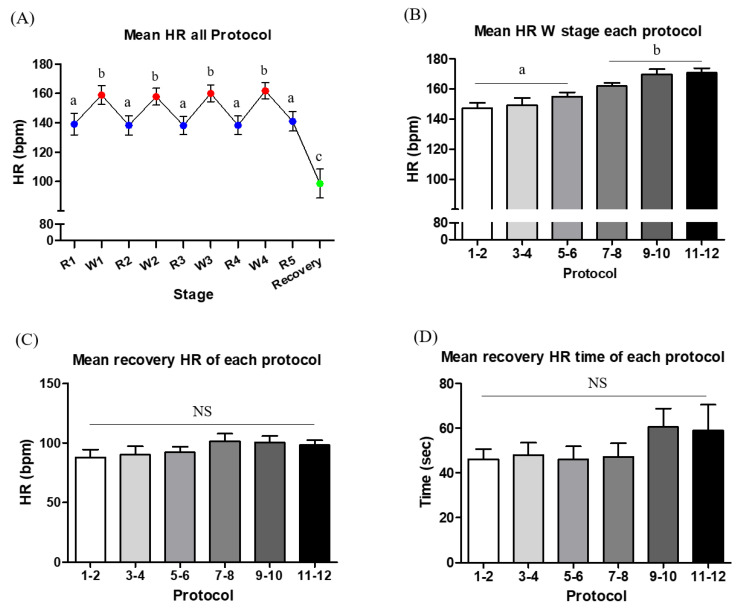
Analysis of the mean heart rate (HR) during exercise and the recovery HR after exercise. (**A**) The mean HR values for all dogs during incomplete resting stage (R1–R5), workout stage (W1–W4), and recovery. (**B**) Changes in the mean HR during the W stage according to treadmill exercise protocol (1–6 vs. 7–12). (**C**) The recovery HR at 1 min after exercise (**D**) The recovery HR time to reach the resting HR after exercise ^a, b, c^ Superscripts indicate significant differences when comparing the average heart rate of each stage or protocol (*p* < 0.05). NS indicates no significant difference.

Figure 3A shows the HR response during interval exercise, which included a series of workout (W) stages and incomplete resting (R) stages. The mean HR during the W stage was significantly higher than that during the R stage (*p* < 0.01). To evaluate if the exercise intensity protocol was progressively overloaded, we compared the mean HR of the W stage in every two protocol intervals. The mean HR during protocols 7–12 was significantly higher than for protocols 1–6 (Figure 3B). The recovery HR at 1 min after exercise (Figure 3C) and the recovery HR time to reach the resting HR (Figure 3D) were not different between protocols, respectively. Additionally, the mean resting HR measured over the entire experiment was 83.8 ± 6.3 bpm, and the resting HR was not different between protocols (data not shown).

### 3.2. Bone Mineral Density and Muscle Volume

BMD in the femur and vertebrae was measured using QCT before and after exercise. Post-exercise femoral BMD (610.2 ± 27.8 HA) increased significantly by 12.6% (*p* < 0.01) compared with pre-exercise BMD (541.6 ± 16.7 HA). In contrast, there was no difference in vertebral BMD, although exercise (317.2 ± 6.6 HA; 291.4 ± 5.4 HA) increased BMD by 8.8%. Moreover, MV values before and after exercise were not significantly different (Table 2).

### 3.3. Hematological and Serum Biochemistry Parameters

Table 3 shows the differences in hematological and serum biochemistry parameters between pre-exercise and post-exercise. The white blood cell and mean corpuscular hemoglobin concentration (MCHC) levels were significantly lower in post-exercise than in pre-exercise. The levels of mean corpuscular volume (MCV), a marker of the average red blood cell size and volume, were significantly increased in post-exercise. TALP, a serum bone marker, showed a significant increase in post-exercise compared with pre-exercise (*p* < 0.01), but calcium and phosphorus levels were not different. Both aspartate transaminase and creatine kinase levels increased significantly in post-exercise. All hematological and serum biochemistry parameters in pre-exercise and post-exercise dogs were within the reference range.

### 3.4. Correlations between Bone Mineral Density, Muscle Volume, and Serum Biochemistry Parameters

**Figure 4:**
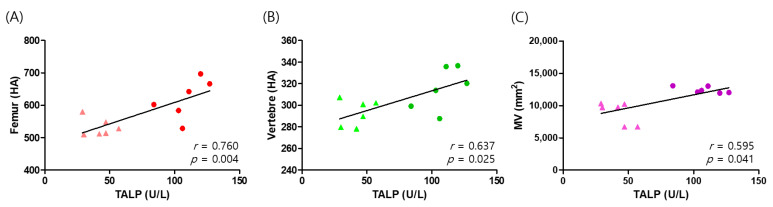
Correlations between total alkaline phosphatase (TALP) and bone mineral density (BMD) and muscle volume (MV) in dogs. (**A**) Correlation between TALP and individual BMD at the femur. (**B**) Correlation between TALP and individual BMD at the vertebrae. (**C**) Correlation between TALP and individual MV. The triangles show pre-exercise TALP samples, and the circles show post-exercise.

Figure 4 illustrates the relationship between TALP and BMD in the femur and vertebrae and MV in the thigh. We found a significant correlation between TALP and BMD in the femur (r = 0.760; *p* = 0.004) and vertebrae (r = 0.637; *p* = 0.025). We also found a positive relationship between TALP and MV (r = 0.595; *p* = 0.041). Those results provide evidence that exercise-induced increases in TALP are associated with increases in BMD and MV.

## 4. Discussion

The main objective of this study was to examine the effects of long-term interval exercise training on HR, BMD, MV, and serum biochemistry parameters in dogs. A primary finding was that the HR response to interval treadmill exercise in different stages and protocols was normal and affirmative. A secondary finding of our study was that interval exercise enhanced BMD in the femur and induced an increment in TALP, aspartate aminotransferase, and creatine kinase biomarkers. To the best of our knowledge, these are the first findings indicating that long-term interval exercise training is feasible for dogs and can improve BMD in the femur. We suggest that increased TALP levels may be an associated mechanism of increasing BMD with exercise in dogs.

Ferasin et al. [34] showed that dogs frequently refuse to exercise on the treadmill and are easily distracted in a laboratory environment. Due to those tendencies, adequate acclimatization is needed before initiating the exercise program. In this study, the beagles did not show any rejection or maladaptive behaviors. In addition, the treadmill interval exercises did not cause any side effects or adverse reactions in healthy dogs. All dogs were able to complete the exercise program and were in good physical condition. Our results are consistent with our previous findings of normal physiological and behavioral responses to treadmill exercise [35]. This may be because the dogs had a sufficient adaptive period on the treadmill, a well-designed exercise program was used, and the study veterinarians and researchers provided adequate animal care. Under these stringent experimental conditions, we aimed to explore the potential effect of long-term interval exercise on HR, BMD, MV, and serum biochemistry parameters in beagles.

The mean HRs of all dogs who performed interval exercise for 12 weeks had changed according to protocol intensity and progress (1–6 week < 7–12 week; *p* <0.05). In addition, following the FITT-VP principle, we were able to identify a regular mean HR change by organizing a suitable exercise program for dogs. Unlike other dog studies that found irregular HR patterns that were not proportional to the activity and external stimulus [36,37], our results showed a normal HR response, which was gradually increased in response to exercise intensity. The reason for such a result seems to be because we created an optimal research environment by providing proper controls to anticipate the dogs’ sensitivities to sounds and odors. Additionally, other studies demonstrated that the abnormal HR responses to exercise, termed chronotropic incompetence, have been shown to be predictive of all-cause mortality and cardiovascular disease in humans [11,38]. Therefore, the interval exercise protocol developed in this study may be applicable for promoting or maintaining cardiovascular health in dogs.

Next, we explored BMD and MV to evaluate whether an adaptive HR response to the exercise training protocol was beneficial to bone and muscle health. Our findings corroborate previous evidence that an interval exercise program that applies the FITT principle and is developed based on former studies can increase BMD [16,17,18,19,20,21,22,23,39,40]. The 12.6% increase in BMD that we observed is consistent with human studies that found an association between BMD and injury [41] or disease [42]. In humans, BMD is a key measure for diagnosing osteoporosis [42], and a 3–5% increase in BMD has been shown to reduce fracture risk by 20–30% [42]. There are few studies examining the effect of exercise on BMD in dogs, and those studies found that aerobic exercise training in dogs resulted in either decreased or unchanged BMD [39,40]. Those different results might be associated with age, sex, and exercise methodology. Puustjarvi et al. [39] suggested that treadmill running exercise would no longer positively affect BMD as a female dog’s growth plates close at 70 weeks of age. In contrast, several human studies confirmed an increase in post-exercise BMD, regardless of age and sex [22]. The cause of this discrepancy is not clear, but the inclusion of intensity as a FITT component might be important because intensity induces a BMD increase and, thus, is a primary influence on the extent of the training effect [43]. Currently, the optimal intensity level for interval exercise to enhance BMD in dogs is not known [39], but in humans, the proper endurance exercise intensity has been estimated to be 55–75% of HR_max_ [44]. In this study, the mean HR during the workout stage was 158.2 bpm, and the dogs continued to exercise for 36 min for 12 weeks. A previous study reported 230 bpm for HR_max_ in their study dogs [10]; thus, the intensity of interval exercise imposed on each dog in this study was approximately 68% of HR_max_. Furthermore, the combination of FITT components with progressive and overloading workouts may be associated with the BMD improvements observed in our study dogs.

The benefits of regular exercise on BMD may be primarily linked to mechanical loading mechanisms [45]. Evidence for the Mechanostat Theory of mechanical loading has been confirmed in several animal studies [46,47]. Rats are tetrapodal animals that are known to have higher tibia stress because the tibia is subjected to greater weight-bearing during treadmill exercise compared to the vertebrae [48]. In a previous study of rats, treadmill exercise increased tibial BMD but not vertebral BMD [49]. Dogs, like rats, are tetrapodal animals, and their femurs are more likely to receive mechanical loads [50] and to be more weight-bearing than the vertebrae when running on treadmills [49]. Our findings are consistent with the results of other animal studies and support the concept that weight-bearing activity has a positive influence on bone health [51].

Many studies have suggested that treadmill exercise improves BMD, but the precise underlying mechanism remains elusive [45]. Here, we examined the effects of treadmill exercise on serum bone markers such as calcium, phosphorus, and TALP to identify biological mechanisms. We found that exercise-induced increases in TALP are associated with increases in BMD. TALP is a critical biomarker to assess BMD accurately and efficiently in the absence of liver disease [52]. Several isoenzymes of TALP exist in various organs besides bone (e.g., liver, kidney), and serum TALP, derived mostly from bones, reflects the sum of those isoenzymes [53]. Particularly in young dogs, changes in TALP result from a bone-specific isoenzyme [54] because 96% of TALP consists of this bone-specific isoenzyme [55]. The bone-specific isoenzyme exists on the plasma membrane of osteoblasts and is carried through systemic circulation during the bone mineralization process [56]. TALP plays a role in the hydrolysis of inorganic pyrophosphate and then generates inorganic phosphate to maintain the appropriate ratio of inorganic pyrophosphate to inorganic phosphate, which is essential for the mineralization process [57]. Therefore, the upregulation of TALP in the two-year-old dogs from this study is considered a positive biomarker associated with increased BMD.

Some human studies suggested potential increases in MV with aerobic exercise and interval exercise [58,59,60]. For example, Harber and his colleagues [58] proved an aerobic exercise-induced increase in MV determined via MRI in young and older men. Malia et al. [61] showed that short-term (3 weeks) high-intensity interval training improved muscle size in vastus lateralis in overweight and obese adults. It has been reported that the increment in MV with high intensity interval training is associated with the activation of the proliferator activated receptor gamma coactivator 1 (PGC-1γ) molecules [62,63]. The activation of these key molecules, which promote mitochondrial biogenesis, substrate transfer, and oxidation capability of skeletal muscles, occurs at high-intensity levels when many fast-twitch muscles are recruited, ultimately causing muscle hypertrophy [60,62,64,65,66,67]. In the present study, we found no significant change in MV with long-term interval exercise. The reasons for the discrepancy between the findings of this study and other human studies are not clear, but the spontaneity and intensity of exercise might be involved. It seems that the dog’s less spontaneous habit of exercising with greater force and the moderate intensity of exercise used in the present study might not be sufficient to increase the MV [68]. Meanwhile, correlation analysis revealed a positive association between TALP and MV in exercised dogs. To our knowledge, this is the first report to identify a significant correlation between TALP and MV in exercised dogs. However, the cause of these consequences is unknown, exercise-induced crosstalk between muscles and bones may be involved, and further research is needed. This study has several limitations. Due to the characteristics of dogs, which are heavy animals, it was difficult to secure the population, so the number of samples was small. Our research has been conducted on only male beagle breeds, so dogs of different sexes and breeds need to be tested. In addition, it was challenging to understand the precise mechanisms for dogs, exercise, and BMD from this study because there was no control group, and no gene or protein analysis related to BMD was performed.

## 5. Conclusions

We demonstrated that interval exercise has a positive impact on BMD in dogs, and exercise-induced enhancement of BMD is associated with increased TALP levels. In addition, this study confirmed that QCT could be used as a measure to assess subtle changes in MV and BMD after a machine-running exercise intervention. Further investigations are needed to determine the impact of exercise on cardiovascular fitness-, bone-, and muscle-related genes in dogs. Such research would improve our understanding of bone-exercise mechanisms and bone-muscle-interaction mechanisms, which would yield fundamental insights into key challenges in exercise science research and the clinical field.

## Figures and Tables

**Table 1:** Characteristics of study dogs.

Parameters (Unit)	Dogs
No. of Dogs	6
Sex	Male ^1^
Age (months)	29.1 ± 6.7 ^2^
Weight (kg)	10.9 ± 0.5

Age and weight data are represented as mean ± SD. ^1^ All dogs were intact. ^2^ Dog ages ranged from a minimum of 21.5 months to a maximum of 35.5 months.

**Table 2:** Analysis of bone mineral density and muscle volume before and after exercise in dogs.

Parameters (Unit)	Pre-Exercise	Post-Exercise	Rate of Increase (%) ^1^
Femur (HA)	541.6 ± 16.7	610.2 ± 27.8 *	12.6
Vertebra (HA)	291.4 ± 5.4	317.2 ± 6.6	8.8
Muscle volume (mm^2^)	5384.0 ± 890.7	5434.0 ± 740.3	0.9

Bone mineral density and muscle volume data are represented as mean ± SEM. * Significant difference between pre-exercise and post-exercise (*p* < 0.05). ^1^ The rate of increase in parameters was calculated as (post-exercise mean—pre-exercise mean) ÷ post-exercise mean × 100.

**Table 3:** Analysis of hematological and serum biochemistry in dogs.

Parameters (Unit)	Pre-Exercise	Post-Exercise	Reference Range
White blood cell (K/μL) ^1^	10,110.0 ± 903.4	7138.0 ± 568.8 *	6000–12,000
Red blood cell (M/μL) ^1^	660.8 ± 10.14	698.8 ± 21.61	570–880
Hemoglobin (g/dL) ^1^	15.5 ± 0.3	16.3 ± 0.6	12.9–18.4
MCV (fL) ^1^	65.7 ± 0.6	67.4 ± 0.6 *	58.8–71.2
MCH (pg) ^1^	23.4 ± 0.1	23.3 ± 0.1	20.5–24.2
MCHC (g/dL) ^1^	35.7 ± 0.3	34.5 ± 0.2 *	31–36.2
Calcium (mg/L) ^2^	9.5 ± 0.2	9.0 ± 0.0	9.0–11.9
Phosphorus (mg/L) ^2^	3.9 ± 0.2	4.0 ± 0.2	1.3–6.3
TALP (U/L) ^2^	68.6 ± 9.2	81.3 ± 17.2 *	0–97.9
ALT (U/L) ^2^	36.3 ± 3.7	41.0 ± 5.0	5.8–83.3
AST (U/L) ^2^	23.5 ± 1.0	33.5 ± 1.6 *	11.7–42.5
BUN (mmol/L) ^2^	14.3 ± 9.3	12.3 ± 0.7	9.6–31.4
Creatinine (mg/L) ^2^	0.7 ± 0.0	0.7 ± 0.0	0.4–1.3
Glucose (mmol/L) ^2^	94.8 ± 3.2	96.3 ± 5.4	74.5–120
Albumin (g/dL) ^2^	3.8 ± 0.1	3.8 ± 0.0	2.6–4.4
Total protein (g/dL) ^2^	6.8 ± 0.2	6.7 ± 0.2	5.7–7.5
Cholesterol (mg/L) ^2^	218.5 ± 23.0	208.0 ± 25.3	112–312
Triglycerides (mmol/L) ^2^	59.8 ± 9.8	63.6 ± 12.8	21–133
Creatine kinase (U/L) ^2^	114.8 ± 5.3	214.0 ± 20.8 *	8–216

MCV: mean corpuscular volume; MCH: mean corpuscular hemoglobin; MCHC: mean corpuscular hemoglobin concentration; ALT: alanine transaminase; AST: aspartate transaminase; BUN: blood urea nitrogen. ^1^ means hematological parameters. ^2^ means serum biochemistry parameters. Serum biochemistry data are represented as mean ± SEM. * Significant difference between pre-and post-exercise measures (*p* < 0.05).

## Data Availability

All data used to support the findings of this study are included within the article. The analyzed data during the current study are available from the corresponding author upon reasonable request.

## Supplementary Materials

The following are available online at https://www.mdpi.com/article/10.3390/ani11092528/s1, Table S1: Adaptive training procedure for dogs..

## References

[1] MacKinnon M. (2010). ‘Sick as a dog’: Zooarchaeological evidence for pet dog health and welfare in the Roman world. World Archaeol..

[2] Bartges J., Kushner R.F., Michel K.E., Sallis R., Day M.J. (2017). One Health Solutions to Obesity in People and Their Pets. J. Comp. Pathol..

[3] Winkle M., Johnson A., Mills D. (2020). Dog Welfare, Well-Being and Behavior: Considerations for Selection, Evaluation and Suitability for Animal-Assisted Therapy. Animals.

[4] Mosier J.E. (1989). Effect of Aging on Body Systems of the Dog. Vet. Clin. N. Am.-Small.

[5] Alansare A., Alford K., Lee S., Church T., Jung H.C. (2018). The Effects of High-Intensity Interval Training vs. Moderate-Intensity Continuous Training on Heart Rate Variability in Physically Inactive Adults. Int. J. Environ. Res. Public Health.

[6] Marzuca-Nassr G.N., Artigas-Arias M., Olea M.A., SanMartin-Calisto Y., Huard N., Duran-Vejar F., Beltran-Fuentes F., Munoz-Fernandez A., Alegria-Molina A., Sapunar J. (2020). High-intensity interval training on body composition, functional capacity and biochemical markers in healthy young versus older people. Exp. Gerontol..

[7] Lee H.S., Oh H.J., Lee S.H., Kim J.W., Kim J.-H. (2019). Comparison of physiological and hematological responses to treadmill exercise in younger and older adult dogs. Korea Inst. Sport Sci..

[8] Piccione G., Casella S., Panzera M., Giannetto C., Fazio F. (2012). Effect of Moderate Treadmill Exercise on Some Physiological Parameters in Untrained Beagle Dogs. Exp. Anim. Tokyo.

[9] Rovira S., Munoz A., Riber C., Benito M. (2010). Heart rate, electrocardiographic parameters and arrhythmias during agility exercises in trained dogs. Rev. Med. Vet. Toulouse.

[10] Radin L., Belic M., Bottegaro N.B., Hrastic H., Torti M., Vucetic V., Stanin D., Vrbanac Z. (2015). Heart rate deflection point during incremental test in competitive agility border collies. Vet. Res. Commun..

[11] Freeman J.V., Dewey F.E., Hadley D.M., Myers J., Froelicher V.F. (2006). Autonomic nervous system interaction with the cardiovascular system during exercise. Prog. Cardiovasc. Dis..

[12] Rizzo M., Arfuso F., Alberghina D., Giudice E., Gianesella M., Piccione G. (2017). Monitoring changes in body surface temperature associated with treadmill exercise in dogs by use of infrared methodology. J. Therm. Biol..

[13] Restan A.Z., Zacche E., da Silva S.B., Cerqueira J.A., Carfiofi A.C., Queiroz-Neto A., Camacho A.A., Ferraz G.C. (2019). Lactate and glucose thresholds and heart rate deflection points for Beagles during intense exercise. Am. J. Vet. Res..

[14] Bilezikian J.P. (1999). Osteoporosis in men. J. Clin. Endocr. Metab..

[15] Kuwabara A., Tanaka K., Tsugawa N., Nakase H., Tsuji H., Shide K., Kamao M., Chiba T., Inagaki N., Okano T. (2009). High prevalence of vitamin K and D deficiency and decreased BMD in inflammatory bowel disease. Osteoporos. Int..

[16] Valimaki V.V., Loyttyniemi E., Valimaki M.J. (2006). Quantitative ultrasound variables of the heel in Finnish men aged 18-20 yr: Predictors, relationship to bone mineral content, and changes during military service. Osteoporos. Int..

[17] Kida K., Osada N., Akashi Y.J., Sekizuka H., Omiya K., Miyake F. (2008). The exercise training effects of skeletal muscle strength and muscle volume to improve functional capacity in patients with myocardial infarction. Int. J. Cardiol..

[18] Laredo J.D., El Quessar A., Bossard P., Vuillemin-Bodaghi V. (2001). Vertebral tumors and pseudotumors. Radiol. Clin. N. Am..

[19] Lester M.E., Urso M.L., Evans R.K., Pierce J.R., Spiering B.A., Maresh C.M., Hatfield D.L., Kraemer W.J., Nindl B.C. (2009). Influence of exercise mode and osteogenic index on bone biomarker responses during short-term physical training. Bone.

[20] Burgomaster K.A., Howarth K.R., Phillips S.M., Rakobowchuk M., MacDonald M.J., Mcgee S.L., Gibala M.J. (2008). Similar metabolic adaptations during exercise after low volume sprint interval and traditional endurance training in humans. J. Physiol. Lond..

[21] Farias L.F., Browne R.A.V., Frazao D.T., Dantas T.C.B., Silva P.H.M., Freitas R.P.A., Aoki M.S., Costa E.C. (2019). Effect of Low-Volume High-Intensity Interval Exercise and Continuous Exercise on Delayed-Onset Muscle Soreness in Untrained Healthy Males. J. Strength Cond. Res..

[22] Alghadir A.H., Aly F.A., Gabr S.A. (2014). Effect of moderate aerobic training on bone metabolism indices among adult humans. Pak. J. Med. Sci..

[23] Bell K.E., Seguin C., Parise G., Baker S.K., Phillips S.M. (2015). Day-to-Day Changes in Muscle Protein Synthesis in Recovery From Resistance, Aerobic, and High-Intensity Interval Exercise in Older Men. J. Gerontol. A Biol..

[24] Rauch F., Tutlewski B., Fricke O., Rieger-Wettengl G., Schauseil-Zipf U., Herkenrath P., Neu C.M., Schoenau E. (2001). Analysis of cancellous bone turnover by multiple slice analysis at distal radius—A study using peripheral quantitative computed tomography. J. Clin. Densitom..

[25] Schoenau E., Neu C.M., Mokov E., Wassmer G., Manz F. (2000). Influence of puberty on muscle area and cortical bone area of the forearm in boys and girls. J. Clin. Endocr. Metab..

[26] De Decker S., Lam R., Packer R.M.A., Gielen I.M.V.L., Volk H.A. (2015). Thoracic and Lumbar Vertebral Bone Mineral Density Changes in a Natural Occurring Dog Model of Diffuse Idiopathic Skeletal Hyperostosis. PLoS ONE.

[27] Chalmers H.J., Dykes N.L., Lust G., Farese J.P., Burton-Wurster N.I., Williams A.J., Todhunter R.J. (2006). Assessment of bone mineral density of the femoral head in dogs with early osteoarthritis. Am. J. Vet. Res..

[28] Mitsiopoulos N., Baumgartner R.N., Heymsfield S.B., Lyons W., Gallagher D., Ross R. (1998). Cadaver validation of skeletal muscle measurement by magnetic resonance imaging and computerized tomography. J. Appl. Physiol..

[29] Woods G., Gunz N.I., Handel I., Liuti T., Mellanby R.J., Schwarz T. (2021). Computed Tomography Osteodensitometry for Assessment of Bone Mineral Density of the Canine Head-Preliminary Results. Animals.

[30] Sutherland-Smith J., Hutchinson D., Freeman L.M. (2019). Comparison of computed tomographic attenuation values for epaxial muscles in old and young dogs. Am. J. Vet. Res..

[31] Gordon-Evans W.J., Johnson A.L., Knap K.E., Griffon D.J. (2019). The effect of body condition on postoperative recovery of dachshunds with intervertebral disc disease treated with postoperative physical rehabilitation. Vet. Surg..

[32] Lee D., Lee Y., Choi W., Chang J., Kang J.H., Na K.J., Chang D.W. (2015). Quantitative CT assessment of bone mineral density in dogs with hyperadrenocorticism. J. Vet. Sci..

[33] Kwon D., Kim J., Lee H., Kim B., Han H., Oh H., Kim M., Yoon H., Lee B., Eom K. (2018). Quantitative computed tomographic evaluation of bone mineral density in beagle dogs: Comparison with dual-energy x-ray absorptiometry as a gold standard. J. Vet. Med. Sci..

[34] Ferasin L., Marcora S. (2007). A pilot study to assess the feasibility of a submaximal exercise test to measure individual response to cardiac medication in dogs with acquired heart failure. Vet. Res. Commun..

[35] Lee H.S., Lee S.H., Kim J.W., Lee Y.S., Lee B.C., Oh H.J., Kim J.H. (2020). Development of Novel Continuous and Interval Exercise Programs by Applying the FITT-VP Principle in Dogs. Sci. World J..

[36] Miyazaki H., Yoshida M., Samura K., Matsumoto H., Ikemoto F., Tagawa M. (2002). Ranges of diurnal variation and the pattern of body temperature, blood pressure and heart rate in laboratory beagle dogs. Exp. Anim. Tokyo.

[37] Beerda B., Schilder M.B.H., van Hooff J.A.R.A.M., de Vries H.W., Mol J.A. (1998). Behavioural, saliva cortisol and heart rate responses to different types of stimuli in dogs. Appl. Anim. Behav. Sci..

[38] Hammond H.K., Kelly T.L., Froelicher V. (1983). Radionuclide imaging correlatives of heart rate impairment during maximal exercise testing. J. Am. Coll. Cardiol..

[39] Puustjarvi K., Karjalainen P., Nieminen J., Arokoski J., Parviainen M., Helminen H.J., Soimakallio S. (1992). Endurance Training Associated with Slightly Lowered Serum Estradiol Levels Decreases Mineral Density of Canine Skeleton. J. Bone Miner. Res..

[40] Puustjarvi K., Lappalainen R., Niemitukia L., Arnala I., Nieminen J., Tammi M., Helminen H.J. (1995). Long-distance running alters bone anthropometry, elemental composition and mineral density of young dogs. Scand. J. Med. Sci. Sports.

[41] Gomez-Bruton A., Matute-Llorente A., Gonzalez-Aguero A., Casajus J.A., Vicente-Rodriguez G. (2017). Plyometric exercise and bone health in children and adolescents: A systematic review. World J. Pediatr..

[42] Berry S.D., Dufour A.B., Travison T.G., Zhu H., Yehoshua A., Barron R., Recknor C., Samelson E.J. (2018). Changes in bone mineral density (BMD): A longitudinal study of osteoporosis patients in the real-world setting. Arch. Osteoporos..

[43] Liang M.T.C., Braun W., Bassin S.L., Dutto D., Pontello A., Wong N.D., Spalding T.W., Arnaud S.B. (2011). Effect of High-Impact Aerobics and Strength Training on BMD in Young Women Aged 20-35 Years. Int. J. Sports Med..

[44] Ghasemalipour H., Eizadi M. (2018). The Effect of Aerobic Training on Some Bone Formation Markers (Osteocalcin, Alkaline Phosphatase) in Asthma Treated with Inhaled Corticosteroids. J. Res. Med. Sci..

[45] Berman A.G., Hinton M.J., Wallace J.M. (2019). Treadmill running and targeted tibial loading differentially improve bone mass in mice. Bone Rep..

[46] Wolff J. (1892). Das gesetz der transformation der knochen. Medicine.

[47] Sun X.L., Li F.B., Ma X.L., Ma J.X., Bin Z., Yang Z., Li Y.J., Lv J.W., Xinmin M.M. (2015). The Effects of Combined Treatment with Naringin and Treadmill Exercise on Osteoporosis in Ovariectomized Rats. Sci. Rep.-UK.

[48] Yeh J.K., Aloia J.F., Chen M.M., Tierney J.M., Sprintz S. (1993). Influence of Exercise on Cancellous Bone of the Aged Female Rat. J. Bone Miner. Res..

[49] Iwamoto J., Takeda T., Ichimura S. (1998). Effects of exercise on bone mineral density in mature osteopenic rats. J. Bone Miner. Res..

[50] Martin R.K., Albright J.P., Clarke W.R., Niffenegger J.A. (1981). Load-Carrying Effects on the Adult Beagle Tibia. Med. Sci. Sport Exerc..

[51] Bennell K.L., Malcolm S.A., Khan K.M., Thomas S.A., Reid S.J., Brukner P.D., Ebeling P.R., Wark J.D. (1997). Bone mass and bone turnover in power athletes, endurance athletes, and controls: A 12-month longitudinal study. Bone.

[52] Rudberg A., Magnusson P., Larsson L., Joborn H. (2000). Serum isoforms of bone alkaline phosphatase increase during physical exercise in women. Calcif. Tissue Int..

[53] Tsuritani I., Honda R., Ishizaki M., Yamada Y., Aoshima K., Kasuya M. (1994). Serum Bone-Type Alkaline-Phosphatase Activity in Women Living in a Cadmium-Polluted Area. Toxicol. Lett..

[54] Stockham S.L., Scott M.A. (2013). Fundamentals of Veterinary Clinical Pathology.

[55] Syakalima M., Takiguchi M., Yasuda J., Hashimoto A. (1997). The age dependent levels of serum ALP isoenzymes and the diagnostic significance of corticosteroid-induced ALP during long-term glucocorticoid treatment. J. Vet. Med. Sci..

[56] Gala J., Diaz-Curiel M., de la Piedra C., Calero J. (2001). Short- and long-term effects of calcium and exercise on bone mineral density in ovariectomized rats. Brit. J. Nutr..

[57] Terkeltaub R.A. (2001). Inorganic pyrophosphate generation and disposition in pathophysiology. Am. J. Physiol. Cell Physiol..

[58] Konopka A.R., Suer M.K., Wolff C.A., Harber M.P. (2014). Markers of Human Skeletal Muscle Mitochondrial Biogenesis and Quality Control: Effects of Age and Aerobic Exercise Training. J. Gerontol. A-Biol..

[59] Harber M.P., Konopka A.R., Undem M.K., Hinkley J.M., Minchev K., Kaminsky L.A., Trappe T.A., Trappe S. (2012). Aerobic exercise training induces skeletal muscle hypertrophy and age-dependent adaptations in myofiber function in young and older men. J. Appl. Physiol..

[60] Blackwell J.E.M., Gharahdaghi N., Brook M.S., Watanabe S., Boereboom C.L., Doleman B., Lund J.N., Wilkinson D.J., Smith K., Atherton P.J. (2021). The physiological impact of high-intensity interval training in octogenarians with comorbidities. J. Cachexia Sarcopenia Muscle.

[61] Blue M.N.M., Smith-Ryan A.E., Trexler E.T., Hirsch K.R. (2018). The effects of high intensity interval training on muscle size and quality in overweight and obese adults. J. Sci. Med. Sport.

[62] Little J.P., Safdar A., Bishop D., Tarnopolsky M.A., Gibala M.J. (2011). An acute bout of high-intensity interval training increases the nuclear abundance of PGC-1 alpha and activates mitochondrial biogenesis in human skeletal muscle. Am. J. Physiol.-Reg. I.

[63] Gurd B.J., Perry C.G.R., Heigenhauser G.J.F., Spriet L.L., Bonen A. (2010). High-intensity interval training increases SIRT1 activity in human skeletal muscle. Appl. Physiol. Nutr. Metab..

[64] Wu Z.D., Puigserver P., Andersson U., Zhang C.Y., Adelmant G., Mootha V., Troy A., Cinti S., Lowell B., Scarpulla R.C. (1999). Mechanisms controlling mitochondrial biogenesis and respiration through the thermogenic coactivator PGC-1. Cell.

[65] Egan B., Carson B., Garcia-Roves P., Chibalin A., Sarsfield F., Barron N., McCaffrey N., Moyna N., Zierath J., O’Gorman D.J.J.P. (2010). Exercise intensity-dependent regulation of PGC-1α mRNA abundance is associated with differential activation of upstream signalling kinases in human skeletal muscle. J. Physiol..

[66] Hoshino D., Kitaoka Y., Hatta H.J.T.J.o.P.F., Medicine S. (2016). High-intensity interval training enhances oxidative capacity and substrate availability in skeletal muscle. J. Phys. Fit. Sports Med..

[67] Wyckelsma V.L., Levinger I., McKenna M.J., Formosa L.E., Ryan M.T., Petersen A.C., Anderson M.J., Murphy R.M. (2017). Preservation of skeletal muscle mitochondrial content in older adults: Relationship between mitochondria, fibre type and high-intensity exercise training. J. Physiol. Lond..

[68] Swanson K.D.J., Harper T.A.M., McMichael M., Fries R.C., Lascola K.M., Chandler C., Schaeffer D.J., Chinnadurai S.K. (2019). Development of a perceived exertion scale for dogs using selected physiologic parameters. J. Small Anim. Pract..

