# Supplementary material for: Effects of Interval Exercise Training on Serum Biochemistry and Bone Mineral Density in Dogs
Source: Animals (Basel). 2021 Aug 28;11(9):2528. doi: 10.3390/ani11092528 (PMC8468388; doi:10.3390/ani11092528)
Supplement: Supplementary file 1 [file animals-11-02528-s001.zip › animals-1290861-supplementary.pdf]

Supplementary Table S1. Adaptive training procedure for dogs.

Goal	Method
Building rapport with researchers	1. Gently petting the neck while looking in the dog's eyes
	2. Playing tug-a-war and walking outside
Adapting to laboratory environment and training	1. Practice wearing a Polar H10 and safety belt
	2. Free roaming around the exercise field
	3. Playing tug-a-war on the exercise field
	4. Walking on a treadmill at low speed (<4.0 km/h; within 10 min)
